# Disruption of Nrf2 Enhances the Upregulation of Nuclear Factor-kappaB Activity, Tumor Necrosis Factor-*α*, and Matrix Metalloproteinase-9 after Spinal Cord Injury in Mice

**DOI:** 10.1155/2010/238321

**Published:** 2010-08-24

**Authors:** Lei Mao, Handong Wang, Liang Qiao, Xiaoliang Wang

**Affiliations:** Department of Neurosurgery, School of Medicine, Jinling Hospital, Nanjing University, Nanjing, Jiangsu 210002, China

## Abstract

Matrix metalloproteinase-9 (MMP-9) plays an important role in the acute periods of spinal cord injury (SCI), and its expression is related to the inflammation which could cause the disruption of the blood-spinal barrier (BBB). Nuclear factor erythroid 2-related factor 2 (Nrf2) is a key transcription factor that plays a crucial role in cytoprotection against inflammation. The present study investigated the role of Nrf2 in upregulating of nuclear factor kappa B (NF-*κ*B) activity, tumor necrosis factor-*α* (TNF-*α*), and MMP-9 after SCI. Wild-type Nrf2 (+/+) and Nrf2-deficient (Nrf (−/−)) mice were subjected to an SCI model induced by the application of vascular clips (force of 10 g) to the dura after a three-level T8-T10 laminectomy. We detected the wet/dry weight ratio of impaired spinal cord tissue, the activation of NF-*κ*B, the mRNA and protein levels of TNF-*α* and MMP-9, and the enzyme activity of MMP-9. Nrf2 (−/−) mice were demonstrated to have more spinal cord edema, NF-*κ*B activation, TNF-*α* production, and MMP-9 expression after SCI compared with the wild-type controls. The results suggest that Nrf2 may play an important role in limiting the upregulation of NF-*κ*B activity, TNF-*α*, and MMP-9 in spinal cord after SCI.

## 1. Introduction

Spinal cord injury (SCI) initiates a series of cellular and molecular cascade events and that a combination of secondary injury factors leads to a progressive neuronal injury [1]. It is generally accepted that, among the secondary injury factors, inflammation is the major one and plays a central role in regulating the pathogenesis of SCI [2, 3]. Attenuation of the early inflammatory response after SCI may limit the extent of tissue injury and the consequent disability [4–6]. The proinflammatory cytokine TNF-*α*, which is upregulated immediately after SCI, can enhance vascular permeability [7]. Hence, TNF-*α* is considered as a proinflammatory cytotoxic cytokine. And TNF-*α* is primarily responsible for initiating the cascade of other cytokines in the classic immune response [6, 8]. Inflammatory cells can release matrix metalloproteinases (MMPs), especially MMP-9, which can penetrate the BBB [9]. MMP-9 is upregulated in leucocytes entering the spinal cord, directly facilitating their extravasation, and promoting the tissue damage that they caused [9, 10]. It might be a vascular permeabilizing factor and is a key determinant in secondary tissue damage after SCI.

Nuclear factor erythroid 2-related factor 2 (Nrf2), a leucine zipper redox-sensitive transcription factor, is a pleiotropic regulator of cell survival mechanisms [11]. Under the normal condition, Nrf2 is sequestered in the cytoplasm by a cytosolic regulatory protein named Keap1. Under condition of oxidative or xenobiotic stress, Nrf2 translocates from the cytoplasm to the nucleus and sequentially binds to a promoter sequence called the antioxidant response element (ARE), resulting in the expression of antioxidant and cytoprotective genes that attenuate tissue injury [12–14].

Recent studies have demonstrated that Nrf2 plays a broad role in modulating acute inflammatory response. Nrf2 plays a protective role in cigarette smoke-induced emphysema [15], dextran sulfate sodium- (DSS-) mediated colitis [16], inflammation-mediated colonic tumorigenesis [17], and allergen-mediated airway inflammation [18]. Our previous studies have shown that Nrf2 played an important role in protecting traumatic brain injury- (TBI-) induced secondary brain injury by regulating inflammatory cytokines and attenuating the pulmonary inflammatory response and NF-*κ*B activation after TBI [12, 19].

We have found that SCI could induce the activation of Nrf2-ARE pathway in spinal cord tissue (data not shown), just as Pomeshchik et al. showed [20]. Therefore, it is reasonable to postulate that Nrf2 plays an important role in limiting the spinal cord inflammatory response after SCI. In the present study, we evaluated the effect of Nrf2 genotype on the activation of NF-*κ*B, TNF-*α*, and MMP-9 in spinal cord after SCI.

## 2. Materials and Methods

### 2.1. Animals

Our experiments were conformed to the Guide for the Care and Use of Laboratory Animals by National Institutes of Health (NIH Publications no. 80-23) and approved by the Animal Care and Use Committee of Nanjing University. Male ICR mice (28–32 g) were housed at 23 ± 1°C in humidity-controlled animal quarters with 12 hours light/dark cycle, and with ad libitum access to food and water throughout the study. Breeding pairs of Nrf2-defcient ICR mice were kindly provided by Dr. Thomas W. Kensler (Johns Hopkins University, Baltimore, Md, USA). Homozygous wild-type Nrf2 (+/+) and Nrf2 (−/−)-deficient mice were generated from inbred heterozygous Nrf2 (+/−) mice [14]. Genotypes of Nrf2 (+/+) and Nrf2 (−/−) mice were confirmed by PCR amplification of genomic DNA isolated from the blood. PCR amplification was carried out by using three different primers, 5′-TGGACGGGACTATTGAAGGCTG-3′ (sense for both genotypes), 5′-CGCCTTTTCAGTAGATGGAGG-3′ (antisense for wild type), and 5′-GCGGATTGACCGTAATGGGATAGG-3′ (antisense for LacZ). Age- and weight-matched adult male mice (6–8 weeks, 28–32 g) were separated into four groups (*n* = 42 per group): group I, sham wild-type (Nrf2 +/+); group II, injured wild-type (Nrf2 +/+); group III, sham-deficient (Nrf2 −/−); group IV, injured-deficient (Nrf2 −/−). The mice of sham and injured groups were subjected to identical anesthetic alone or experimental SCI, respectively.

### 2.2. Experiment Protocol

The mouse compression model of SCI established as described previously used to study spinal cord injury in mice [21]. Briefly, the animals were anesthetized with sodium pentobarbital (50 mg/kg, ip). A longitudinal skin incision was made to expose the spine between T8 and T10 vertebral body levels, and the paravertebral muscles of the thoracic-level (T8–T10) vertebrae were removed. Laminectomy was performed at the T8–T10 level, using an operating microscope (M500-N, LAICA, Germany). In sham-operated mice, the muscles and skin wound was then closed. In order to cause an acute-compression injury, we used a vascular clip (with 10 g force, Kent Scientific Corporation, INS 14120, USA) to injure the cord at T9 by bilateral compression for 1 minute. After surgery, the animals were given a subcutaneous saline injection (1-2 mL) immediately. They were left to recover on a warm pad until thermoregulation and the righting reflex was re-established. Then they were returned to their cages with free food and water. The rectal temperature was monitored and was kept at 37 ± 0.5°C (with physical cooling if required) throughout the experiment. As described, animals received manual bladder expression twice daily until urinary retention was relieved [1].

At 24 hours following sham operation or SCI, thirty mice in each group were sacrificed for spinal cord segments collection to assay NF-*κ*B activity, TNF-*α*, and MMP-9. Briefly, mice were deeply anaesthetized with pentobarbital sodium (80 mg/kg) and were transcardially perfused with cold saline (4°C), and then the tissue segments containing the lesion (1 cm on each side of the lesion) were rapidly removed and stored at liquid nitrogen immediately. Six mice of each group were sacrificed at 48 hours after operation for assays of spinal cord water content of spinal cord segments. The last six mice in each group were evaluated by histopathology at 24 hours after SCI. They were perfused with cold saline (4°C), followed by 4% neutral-buffered formalin. Spinal cord segments containing the lesion (0.5 cm on each side of the lesion) were taken, stored overnight in 4% neutral-buffered formalin, then were paraffin embedded, and cut into 5 *μ*m-thick sections. Tissue sections (thickness 5 *μ*m) were deparaffinized with xylene, stained with Hematoxylin/Eosin (H&E), and studied using light microscopy (ECLIPSE E100, Nikon, Japan).

### 2.3. Spinal Cord Water Content

The spinal cord water content was measured as previously described [22]. At 48 hours after SCI, spinal cords were removed from T7 to T11 vertebral body levels, weighed, heated at 98°C for 48 hours, and reweighed. Percent water content was calculated as [(wet weight – dry weight)/wet weight].

### 2.4. Light Microscopy

The segments of each spinal cord tissue were evaluated by an experienced histopathologist. Damaged neurons were counted and the histopathologic changes of the gray matter were scored on a 6-point scale [23, 24]: (0), no lesion observed; (1), gray matter contained 1 to 5 eosinophilic neurons; (2), gray matter contained 5 to 10 eosinophilic neurons; (3), gray matter contained more than 10 eosinophilic neurons; (4), small infarction (less than one-third of the gray matter area); (5), moderate infarction (one-third to one-half of the gray matter area); (6), large infarction (more than half of the gray matter area). The scores of all the sections from each spinal cord were averaged to give a final score for an individual mouse. All the histological studies were performed in a blinded fashion.

### 2.5. Nuclear Protein Extract and EMSA

Spinal cord segments were dissected as previously described and immediately frozen in liquid nitrogen. Nuclear protein extraction followed the method described by Jin et al. in our laboratory [25]. Protein concentration was determined using a bicinchoninic acid assay kit with boving serum albumin as the standard (Pierce Biochemicals, Rockford, Ill, USA).

 EMSA was performed using a commercial kit (Gel Shift Assay System; Promega, Madison, Wis, USA) following the methods described in detail elsewhere [26]. Consensus oligonucleotide probe (5′-AGTTGAGGGGACTTTCCCAGGG-3′) was end labeled with [*γ*-^32P^]-ATP (Free Biotech., Beijing, China) with T4-polynucleotide kinase. Nuclear protein (20 *μ*g) was preincubated in a total volume of 20 *μ*L in a binding buffer, consisting of 10 mmol/L Tris-HCl (PH 7.5), 1 mmol/L MgCl_2_, 0.5 mmol/L NaCl, 4% glycerol, 0.5 mmol/L EDTA, 0.5 mmol/L DTT, and 2 *μ*g poly dI-dC for 20 minutes at room temperature. After addition of the 1 *μ*l ^32^P-labled oligonucleotide probe, the incubation was continued for 20 minutes at room temperature. After adding 1 *μ*l of gel loading buffer, the reaction was stopped, and the mixture was resolved by electrophoresis on 4% nondenaturing polyacrylamide gel in 0.5×TBE buffer (Tris-borate-EDTA) at 390 V for 1 hour at 4°C. The gel was dried and exposed to X-ray film ( Fuji Hyperfilm, Tokyo, Japan) at −70°C with an intensifying screed. Levels of NF-*κ*B DNA binding activity were quantified by computer-assisted densitometric analysis.

### 2.6. Reverse Transcriptional-Polymerase Chain Reaction (RT-PCR)

The levels of TNF-*α* and MMP-9 mRNA expression were determined by RT-PCR. Total RNA was extracted from mouse spinal cord segments with EZgene Tissue RNA Miniprep Kit (Biomiga, Inc., San Diego, CA, USA) according to the manufacturer's instructions. The cDNA was synthesized using Reverse Transcription System (Promega Corporation, Madison, WI, USA) and oligo dT from 2 *μ*g of total RNA. The cDNA was then amplified by PCR using various primer sets as follows: TNF-*α*, 5′-ACGGCATGGATCTCAAAGAC-3′, and 5′-GGTCACTGTCCCAGCATCTT-3′; MMP-9, 5′-CTACTCTGAAGACTTGCCG-3′, and 5′-CCATACAGTTTATCCTGGTC-3′; *β*-actin, 5′-AGTGTGACGTTGACATCCGTA-3′, and 5′-GCCAGAGCAGTAATCTCCTTCT-3′ [19, 27]. PCR products were detected by agarose gel electrophoresis in 2% NuSieve agarose gels (FMC, USA) and visualized by ethidium bromide staining. The intensity of the bands was quantified using ImageJ program, and the ratios of each gene product to *β*-actin product were used as indices of TNF-*α* and MMP-9 mRNA expression.

### 2.7. Enzyme-Linked Immunosorbent Assay (ELISA)

TNF-*α* protein levels were both detected by ELISA at 24 hours after SCI. Portions of spinal cord tissues were homogenized as previously described in PBS containing 2 mmol/L of phenylmethylsulfonyl fluoride (PMSF, Sigma Chemical Co.), 1 mg/L pepstatin A, 1 mg/L aprotinin, 1 mg/L leupeptin, and phosphate-buffered saline solution (pH 7.2) and centrifuged at 12,000 ×g for 20 minutes at 4°C. The supernatant was then collected and total protein was determined by using the Bradford method. TNF-*α* protein was quantified using ELISA kits specific for mouse according to the manufacturers' instructions (Diaclone Research, France) and a previous study of our laboratory [19]. The cytokine TNF-*α* content in the spinal cord samples was expressed as pg per milligram total protein.

### 2.8. Western Blot Analysis for MMP-9

Spinal cord tissues were homogenized in RIPA buffer (1% NP40, 0.5% sodium deoxycholate, 0.1% SDS, 1 mM EDTA, 1 mM EGTA, 1 mM Na_3_VO_4_, 20 mM NaF, 0.5 mM DTT, 1 mM PMSF, and protease inhibitor cocktail in PBS pH 7.4). The homogenate was centrifuged at 1050 ×g (4°C) for 10 min, and microsomal fraction was subsequently centrifuged at 17,000 ×g (4°C) for 20 minutes [28]. The supernatant was collected to evaluate MMP-9 expression. Protein concentrations were determined by using the Bradford method. Fifty micrograms of the resulting cytosolic protein extracts were heat denatured in Laemmli sample loading buffer, separated by 10% sodium dodecyl sulfate polyacrylamide gel electrophoresis, and electrotransferred onto a nitrocellulose membrane. For immunoblotting, membranes were blocked with 5% nonfat dry milk in saline buffer overnight, at 4°C, and then incubated with primary anti-MMP-9 antibody (1 : 500, Calbiochem, Darmstadt, Germany) for 2 hours at room temperature in 1×PBS, 5% w/v nonfat dry milk, and 0.1% Tween-20 (PMT). The membranes were washed three times for 5 minutes in PMT and the secondary incubations were performed with horseradish peroxidase-linked antirabbit IgG (1 : 5000, Jackson ImmunoResearch, West Grove, PA, USA). Positive signals were visualized using enhanced chemiluminescence (ECL; Amersham Biosciences, Bucks, United Kingdom), and serial exposures were made to radiographic film (Fuji Hyperfilm, Tokyo, Japan). Densitometric analysis of the blots was performed with the image analysis program ImageJ. To ascertain that blots were loaded with equal amounts of proteic lysates, membranes were incubated with stripping buffer (100 mM *β*-mercaptoethanol, 2% SDS, 62.5 mM Tris-HCl, pH 6.7) at 50°C for 30 minutes, then washed, blocked, and reprobed overnight at 4°C with mouse anti-*β*-actin monoclonal antibody (1 : 5000; Sigma).

### 2.9. Gelatin Zymography

We used gelatin zymography analysis to show MMP-9 activity of spinal cord tissue at 24 hours after SCI. Samples of spinal cord, prepared from the epicenter, were quickly frozen at −80°C. Each sample was weighed and homogenized (1 : 4 w/v) in lysis buffer containing 50 mM Tris-HCl, pH 7.4, 150 mM NaCl, 5 mM CaCl_2_, 0.2 mM NaN_3_, and 0.01% Triton. Soluble and insoluble extracts were separated by centrifugation and stored at −20°C. The supernatant was collected and total protein was determined using the Bradford method. Equal amounts of the supernatant were analyzed by gel zymography as described previously on 10% SDS-polyacrylamide gels copolymerized with substrate (1 mg/mL gelatin) [29]. The proteins were renatured by incubation in 2.5% Triton X-100 and then incubated in substrate buffer (50 mM Tris-HCl, pH 8.5, 5 mM CaCl_2_) for 24–36 hours at 37°C to enable the MMP-9 to cleave the gelatin. After rinsing in water, each gel was stained with Coomassie blue for 4 hours and destained in 50% methanol. Proteolytic activities were detected by clear bands indicating the lysis of the substrate. Quantification of MMP-9 band density was carried out using the image analysis program ImageJ.

### 2.10. Statistical Analysis

Software SPSS 16.0 was used for the statistical analyses. The results were analyzed by one-way ANOVA followed by a Student-Newman-Keuls test for multiple comparisons. Data are presented as mean ± SE. A *P*-value less than  .05 was considered significant.

## 3. Results

### 3.1. Spinal Cord Tissue Water Content

**Figure 1:**
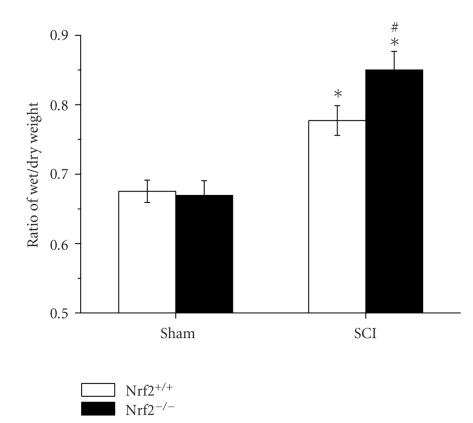
Spinal cord tissue water content in sham and injured Nrf2 (+/+) and Nrf2 (−/−) mice. The figure indicates that the spinal cord water content was significantly increased and was higher in Nrf2 (−/−) mice than that in Nrf2 (−/−) mice after SCI. Bars represent mean ± S.E. (*n* = 6, each group). *P < .01 versus genotype-matched sham-operated mice. ^#^ *P* < .05 versus treatment-matched Nrf2 (+/+) mice.

Similar water content of spinal cord tissue was detected in both sham-operated Nrf2 (+/+) and Nrf2 (−/−) mice. At 48 hours after SCI, water content of spinal cord significantly increased in both injured Nrf2 (+/+) and Nrf2 (−/−) mice compared with the sham-operated mice, respectively. However, the pathological change was severer in Nrf2 (−/−) mice than that in Nrf2 (+/+) mice (0.85 ± 0.03 versus 0.78 ± 0.02, *P* < .05, Figure 1).

### 3.2. Histological Score

**Figure 2:**
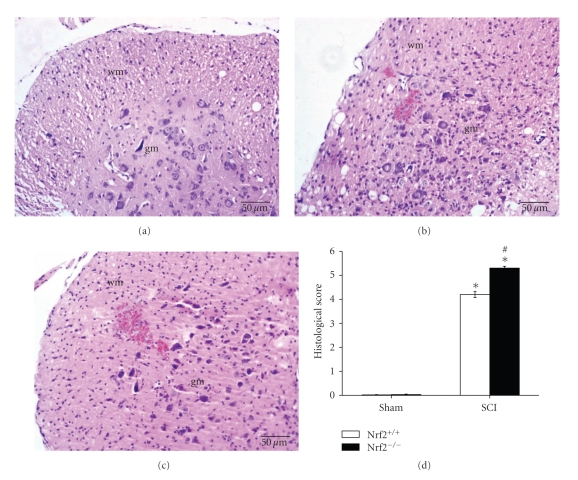
Influence of Nrf2 genotype on histological alterations of the spinal cord tissue 24 hours after SCI. No histologic alteration was observed in spinal cord tissues obtained from sham-operated mice (Figure 2(a)). A significant damage to the spinal cord from SCI mice at the perilesional, as assessed by the presence of edema as well as an alteration of the white matter (Figure 2(b)). Notably, the histological injury was severer in Nrf2 (−/−) mice (Figure 2(c)) than that in Nrf2 (+/+) mice (Figures 2(b) and 2(d)) at 24 hours after SCI. Bars represent mean ± S.E. (*n* = 6, each group). *P < .01 versus genotype-matched sham-operated mice. ^#^ *P* < .01 versus treatment-matched Nrf2 (+/+) mice. wm: white matter; gm: gray matter.

The degree of the spinal cord injury at the perilesional area was evaluated at 24 hours after SCI. The histological score was made by an independent observer. Significant damage to the spinal cord was observed in the SCI mice than that in the sham-operated mice in both Nrf2 (+/+) and Nrf2 (−/−) mice. Notably, the histological injury was severer in Nrf2 (−/−) mice than that in Nrf2 (+/+) mice at 24 hours after SCI (5.3 ± 0.08 versus 4.2 ± 0.13, *P* < .01, Figure 2).

### 3.3. EMSA for NF-*κ*B

**Figure 3:**
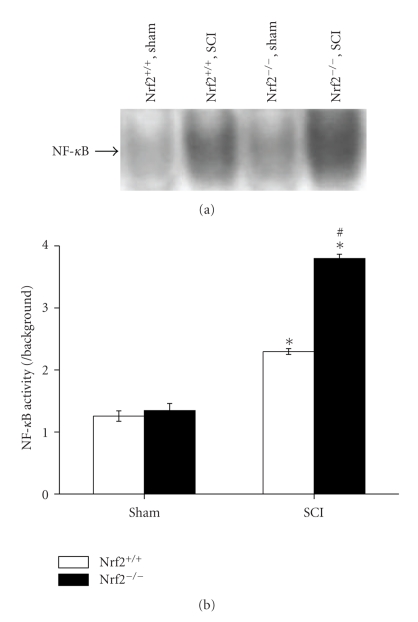
NF-*κ*B activity in the spinal cord segments of sham and injured Nrf2 (+/+) and Nrf2 (−/−) mice. (a) Representative X-ray film shows NF-*κ*B DNA binding activity by EMSA at 24 h after SCI in sham and injured Nrf2 (+/+) and Nrf2 (−/−) mice. (b) Quantification of NF-*κ*B DNA-binding activity was performed by densitometric analysis. The figure indicates that spinal cord NF-*κ*B activity was significantly increased after SCI and was greater in Nrf2 (−/−) mice than in Nrf2 (+/+) mice. Data represents mean ± S.E. (*n* = 6 per group). *P < .01 versus sham control of the same genotype.  *P* < .01 versus injured wild-type mice.

NF-*κ*B activation at 24 hours after SCI was assessed by EMSA. As shown in Figure 3, low NF-*κ*B banding activity was detected in sham-operated mice of both genotypes. SCI induced activation of NF-*κ*B in the spinal cord in both Nrf2 (+/+) and Nrf2 (−/−) mice. Nrf2 (−/−) mice showed an increased susceptibility to SCI-induced activation of NF-*κ*B than their wild-type Nrf2 (+/+) counterparts (3.80 ± 0.07 versus 2.30 ± 0.05, *P* < .01, Figures 3(a) and 3(b)).

### 3.4. RT-PCR for mRNA Levels of TNF-*α* and MMP-9

**Figure 4:**
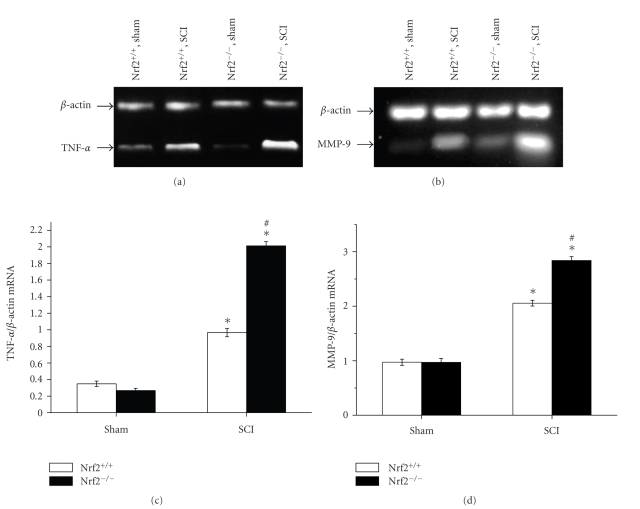
RT-PCR analysis for TNF-*α* and MMP-9 mRNA levels in the spinal cord samples of sham and injured Nrf2 (+/+) and Nrf2 (−/−) mice. (a) and (c) Representative agarose gel images show TNF-*α* and MMP-9 mRNA levels in sham and injured Nrf2 (+/+) and Nrf2 (−/−) mice, respectively. (b) and (d) the graphs show that higher mRNA expression levels of TNF-*α* and MMP-9 were found in Nrf2 (−/−) mice than in Nrf2 (+/+) mice at 24 hours after SCI, respectively. Bars represent mean ± S.E. (*n* = 6, each group). *P < .01 versus genotype-matched sham-operated mice. ^#^ *P* < .01 versus treatment-matched Nrf2 (+/+) mice.

The mRNA levels of two inflammatory gene products, TNF-*α* and MMP-9, were measured by RT-PCR. Similar mRNA expression levels of them were detected in the spinal cord samples of both sham-operated groups. At 24 hours after SCI, increased mRNA expression levels in spinal cord, including TNF-*α* and MMP-9, were detected in both injured Nrf2 (+/+) and Nrf2 (−/−) mice compared with the sham-operated mice, respectively. Higher mRNA expression levels of TNF-*α* were found in Nrf2 (−/−) mice than in Nrf2 (+/+) mice (2.01 ± 0.05 versus 0.97 ± 0.05, *P* < .01, Figures 4(a) and 4(b)). Similarly, significant difference of MMP-9 mRNA level was also observed between Nrf2 (−/−) mice and Nrf2 (+/+) mice (0.99 ± 0.07 versus 0.78 ± 0.04, *P* < .01, Figures 4(c) and 4(d)).

### 3.5. ELISA for TNF-*α*

**Figure 5:**
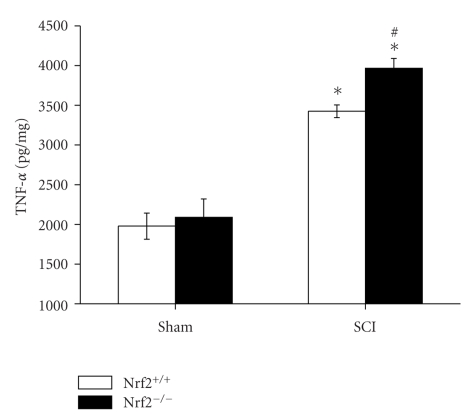
Protein expression levels of TNF-*α* in spinal cord samples of sham and injured Nrf2 (+/+) and Nrf2 (−/−) mice. The figure indicates that spinal cord contents of TNF-*α* were significantly increased and were higher in Nrf2 (−/−) mice than in Nrf2 (+/+) mice at 24 hours after SCI. Bars represent mean ± S.E. (*n* = 6, each group). *P < .01 versus genotype-matched sham-operated mice. ^#^ *P* < .05 versus treatment-matched Nrf2 (+/+) mice.

The protein expression level of TNF-*α* in the spinal cord samples of both sham-operated Nrf2 (+/+) and Nrf2 (−/−) mice was also similar. At 24 h after SCI, higher spinal cord tissue protein expression levels of TNF-*α* were detected in both injured Nrf2 (+/+) and Nrf2 (−/−) mice compared with their respective sham-operated mice. Similarly, higher protein expression levels of TNF-*α* were found in Nrf2 (−/−) mice than in Nrf2 (+/+) mice (3966.60 ± 122.67 versus 3424.04 ± 79.66, *P* < .05, Figure 5).

### 3.6. Western Blot Analysis for MMP-9

**Figure 6:**
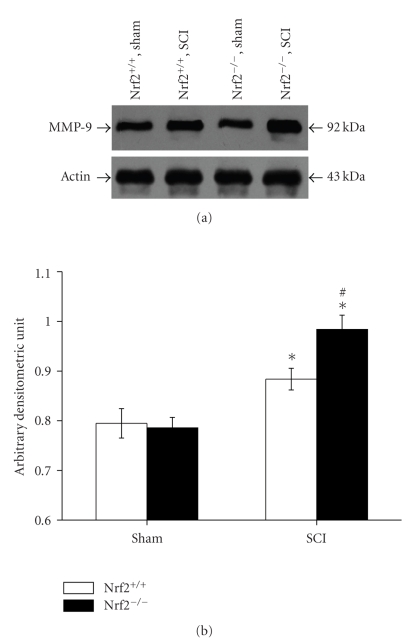
MMP-9 expression by Western blot analysis in spinal cord homogenates from sham and injured Nrf2 (+/+) and Nrf2 (−/−) mice. (a) Representative X-ray film shows MMP-9 expression by Western blot analysis at 24 hours after SCI in sham and injured Nrf2 (+/+) and Nrf2 (−/−) mice. (b) The figure indicates that higher MMP-9 expression levels were found in Nrf2 (−/−) mice than in Nrf2 (+/+) mice at 24 hours after SCI. Bars represent mean ± S.E. (*n* = 6, each group). *P < .01 versus sham control of the same genotype. ^#^ *P* < .01 versus injured wild-type mice.

There is no difference on MMP-9 expression in the spinal cord samples between sham-operated Nrf2 (+/+) and Nrf2 (−/−) mice, as shown by the immunoreactive band migrating at ~92 kDa. At 24 hours after SCI, significant upregulation of MMP-9 expression levels was detected in both injured Nrf2 (+/+) and Nrf2 (−/−) mice compared with their respective sham-operated mice. Higher MMP-9 expression levels were found in Nrf2 (−/−) mice than in Nrf2 (+/+) mice (0.98 ± 0.03 versus 0.88 ± 0.02, *P* < .01, Figures 6(a) and 6(b)).

### 3.7. Gelatin Zymography Analysis for MMP-9

**Figure 7:**
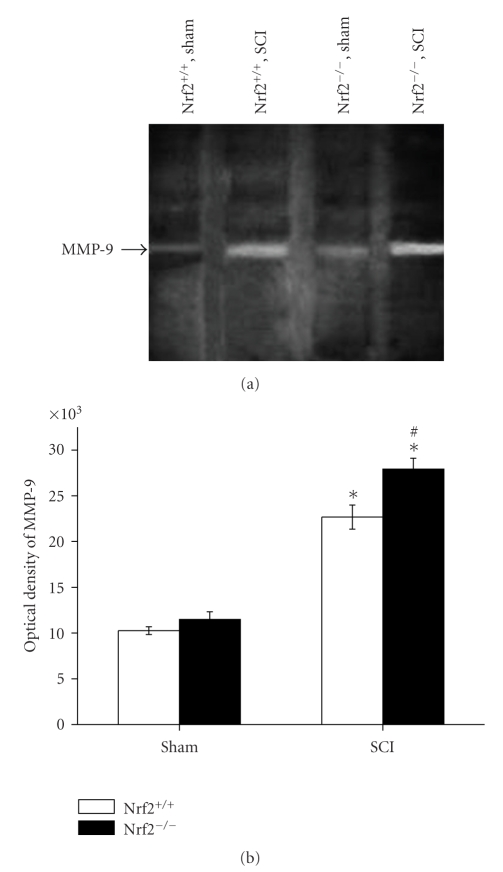
Gelatin zymography analysis for the activity of MMP-9 in spinal cord tissue of sham and injured Nrf2 (+/+) and Nrf2 (−/−) mice. (a) Representative gelatin images show the activity of MMP-9 in sham and injured Nrf2 (+/+) and Nrf2 (−/−) mice. (b) The graphs show that higher activity of MMP-9 was found in Nrf2 (−/−) mice than in Nrf2 (+/+) mice at 24 hours after SCI. Bars represent mean ± S.E. (*n* = 6, each group). *P < .01 versus genotype-matched sham-operated mice. ^#^ *P* < .01 versus treatment-matched Nrf2 (+/+) mice.

The activity of MMP-9 in spinal cord tissue was measured by gelatin zymography analysis. In the spinal cord samples of both sham-operated Nrf2 (+/+) and Nrf2 (−/−) mice, the activity of MMP-9 was similar. At 24 h after SCI, higher activity of MMP-9 was detected in both injured Nrf2 (+/+) and Nrf2 (−/−) mice compared with their respective sham-operated mice, while the activity change was severer in Nrf2 (−/−) mice than in Nrf2 (+/+) mice (27922.79 ± 1185.68 versus 22666.08 ± 1316.41, *P* < .01, Figures 7(a) and 7(b)).

## 4. Discussion

In this study, we found that the mice lacking Nrf2 function have significantly enhanced secondary spinal cord injury characterized by increased spinal cord edema and severer inflammatory response. Increased SCI-induced NF-*κ*B activation and expression of the inflammatory cytokines, TNF-*α* and MMP-9, were observed in the spinal cord of Nrf2 (−/−) mice compared to Nrf2 (+/+) mice. In addition, higher level of MMP-9 activity was found in the spinal cord of Nrf2 (−/−) mice than that in Nrf2 (+/+) mice after SCI. To our knowledge, the findings in this study suggest for the first time that Nrf2 plays a protective role against SCI-induced second spinal cord injury, possibly by limiting the inflammatory response in the spinal cord after SCI.

Activation of NF-*κ*B signaling pathway has been shown to be central to the pathophysiology of spinal cord inflammatory response induced by SCI, and NF-*κ*B can be activated by lesion-induced oxidative stress and cytokines [6, 30]. The functional importance of NF-*κ*B in inflammation is based on its ability to regulate the promoters of multiple inflammatory genes [31]. 

Animal SCI models have shown to have infiltration of inflammatory cells, endothelial activation and injury leading to increased vascular permeability, edema formation, and accumulation of inflammatory mediators [32]. Microvascular endothelial permeability is related to focal adhesive bond and activation of MMPs [33]. MMP-9 is produced by neutrophils and endothelia, facilitating leukocytes diapedesis which may be a vascular permeabilizing factor [34]. In spinal cord injury, MMPs, including MMP-9, contribute to early secondary pathogenesis by disrupting the blood spinal cord barrier and promoting inflammation [35]. 

TNF-*α* is a major initiator of inflammation and is released early after an inflammatory stimulus. It is upregulated immediately after SCI and it can enhance vascular permeability. Evidence has been provided that TNF-*α* induces the production of matrix metalloproteinase MMP-9 [36]. There is evidence that TNF-*α* also plays an important role in the recruitment of inflammatory and immune cells to the injured site. Moreover, the infiltration of immune and inflammatory cell to SCI sites is a major contributor to secondary degeneration [37]. Consistent with other studies, we show that TNF-*α* and MMP-9 were also upregulated during SCI, spinal cord water content at the injury site increased at 2 days after SCI, and significant damage to the spinal cord was observed in the tissue from SCI mice [9, 22, 24]. 

Nrf2 has been reported to be the key regulator of the expression of a group of antioxidant and detoxification enzymes and is considered to play an important role in modulating inflammation in a variety of experimental models [38, 39]. Also, the Nrf2 signaling pathway has been shown to modulate NF-*κ*B activation and expression of NF-*κ*B-dependent genes including MMP-9 [40]. In our past studies, the results suggested that Nrf2 plays an important role in protecting TBI-induced secondary brain injury, possibly by limiting the cerebral upregulation of NF-*κ*B activity, proinflammatory cytokine after TBI [25]. Recently, Pomeshchik et al. showed that SCI could induce Nrf2-ARE pathway activation in spinal cord [20], just as our laboratory has found (data not shown). Also in vitro, phase II enzyme inducers can promote the expression of antioxidative enzymes through Nrf2 and show neuroprotective potential on motor neuron survival in traumatic spinal cord injury [41]. In the present study, Nrf2 (−/−) mice were shown to have severer spinal cord edema, more NF-*κ*B activity, more inflammatory cytokine TNF-*α*, and MMP-9 expression in spinal cord after SCI compared with their wild-type Nrf2 (+/+) counterparts. Taken together, these studies showed that Nrf2 may play an important protective role in limiting the spinal cord inflammatory response after SCI. 

Although numerous in vivo studies have reported that Nrf2 plays a critical role in counteracting the inflammation in a variety of experimental models [15, 16, 25], the precise mechanism underlying this network is still unclear. Several lines of evidence indicate that Nrf2 interferes with inflammatory signaling pathways by inhibiting NF-*κ*B activation through the maintenance of cellular redox status. Oxidative stress from reactive oxygen species (ROS) was considered as a major pathway of secondary injury in SCI [42]. Activation of the NF-*κ*B signaling pathway has been shown to be responsive to excess ROS and is important in the generation of inflammation [39]. Nrf2 has been shown to play an important role in limiting ROS levels and thereby affects the redox-sensitive NF-*κ*B signaling pathway involved in inflammation [14, 40]. Augmentation of cellular antioxidative or detoxification systems via activation of Nrf2-regulated enzymes results in decreased inflammatory cytokine production via inactivation of NF-*κ*B [12]. This constitutes a possible anti-inflammatory mechanism for the attenuated inflammatory response and tissue damage seen in spinal cord from Nrf2 (+/+) mice but not Nrf2 (−/−) mice after SCI. Additional research is necessary to elucidate the entire mechanisms involved in these complicated networks.

## 5. Conclusions

In conclusion, the present study showed that Nrf2 (−/−) mice are more susceptible to SCI-induced spinal cord edema, NF-*κ*B activity, inflammatory TNF-*α* production, and MMP-9 expression, which then contributed to exacerbated spinal cord injury after SCI. These findings raise the possibility that Nrf2 might be a new therapeutic target for the treatment of SCI.
